# Repurposing DPP-4 Inhibitors for Colorectal Cancer: A Retrospective and Single Center Study

**DOI:** 10.3390/cancers13143588

**Published:** 2021-07-17

**Authors:** Lui Ng, Dominic Chi-Chung Foo, Carlos King-Ho Wong, Abraham Tak-Ka Man, Oswens Siu-Hung Lo, Wai-Lun Law

**Affiliations:** 1 Department of Surgery, Li Ka Shing Faculty of Medicine, The University of Hong Kong, Pokfulam, Hong Kong; ccfoo@hku.hk (D.C.-C.F.); tkman@hku.hk (A.T.-K.M.); oswens@hku.hk (O.S.-H.L.); 2 Department of Pharmacology and Pharmacy, Li Ka Shing Faculty of Medicine, The University of Hong Kong, Pokfulam, Hong Kong; carlosho@hku.hk

**Keywords:** colorectal cancer, DPP4, CD26, DPP4-inhibitor, gliptin, immune cell, drug repurposing

## Abstract

**Simple Summary:**

Colorectal cancer is one of the most common causes and the leading cause of cancer deaths worldwide. Its poor prognosis highlights the urgent need for more effective treatments. Repurposing approved drugs is a promising strategy as preclinical screenings can be minimized. The aim of our retrospective study was to investigate the potential of Dipeptidyl-peptidase 4 (DPP4)-inhibitors, which are safe Food and Drug Association (FDA)-approved drugs for treating diabetes, in treating CRC patients. Our findings conclude that CRC patients with diabetes and treated with DPP4-inhibitors in our hospital during 2006–2015, their 5-year prognosis following curative resection was significantly better than those treated with metformin. We further showed that their prognosis was associated with immune cell population features that associated with better prognosis, and immune cell profile is a biomarker for predicting the prognosis of DPP4-inhibitors treated CRC patients.

**Abstract:**

Background: There have been studies reporting the crucial roles of Dipeptidyl-peptidase 4 (DPP4) in colorectal cancer (CRC) initiation and progression, whereas DPP4-inhibitors are safe Food and Drug Association (FDA)-approved drugs for treating diabetes. This study aims to investigate the association between DPP4-inhibitor treatment and the prognosis of CRC patients. Methods: Clinical data of CRC patients with diabetes and the prescription of DPP4-inhibitors who had undergone curative surgery in our hospital between January 2006 and December 2015 were retrieved. Their survival data and immune cell population in circulatory blood were compared to those treated with metformin. Results: The DPP4-inhibitor patient group showed a significantly better 5-year disease-free survival (median DFS = 1733 days, 95% CI = 1596 to 1870 days) when compared to the metformin group (*p* = 0.030, median DFS = 1382 days, 95% CI = 1246 to 1518 days). 33 out of the 92 patients in the metformin group showed recurrence whereas only 3 of the 26 patients in the DPP4-inhibitor group showed recurrence (*p* = 0.033). Cox regression analysis demonstrated that DPP4-inhibitor application is a favorable factor associated with a lower risk of recurrence (Hazard ratio = 0.200, *p* = 0.035). Furthermore, our results suggested that the immune cell profile of CRC patients is a potential biomarker for response to DPP4-inhibitor treatment. Conclusion: This study demonstrated the association of DPP4-inhibitor treatment with a better prognosis of CRC patients.

## 1. Introduction

Colorectal cancer (CRC) is the commonest and the second leading cause of cancer deaths in Hong Kong, being devastative to both diseased individuals and their families. The quality of life and survival time of CRC patients are unfavorable in CRC patients due to the high mortality rate, late diagnosis due to asymptomatic development, metastasis of the tumor, poor effectiveness of treatments and adverse side effects caused by therapies. Current standard CRC treatments include surgery, chemotherapy, radiotherapy, or combinations of them. Although there is recent evidence on the improvement of the overall survival and disease-free survival in CRC, the 5-year survival rate remains below 65% in developed countries and under 50% in developing countries [1]. Moreover, the 5-year relative survival rate was further reduced in colorectal cancer patients with distant metastases (14%–15%) [2]. The poor prognosis for patients, especially individuals with advanced-stage, highlights the urgent need for more effective treatments. Tremendous gains have been made in recent years in achieving long-term responses using immune checkpoint inhibitors (ICIs) for solid tumors such as melanoma, non-small-cell lung cancer (NSCLC) and renal cell cancer [3]. However, most CRCs and metastases of CRC do not exhibit deficient MisMatch Repair (dMMR), and hence a majority of CRC patients do not potentially benefit from immunotherapy [4]. There remains an urgent need to develop better therapeutic targets for CRC patients. Repurposing already approved drugs is a promising strategy because preclinical screenings can be minimized, and there are comparatively lower costs, lower risks and less time.

The role of Dipeptidyl Peptidase 4 (DPP4) inhibitors has been widely studied in cancer-related research. Also known as gliptins, DPP4-inhibitors are a class of oral hypoglycemic drugs that antagonize the enzyme DPP4 and are generally used as a treatment for diabetes mellitus type 2 (DM-II) [5]. The inhibitory effect against DPP4 would promote incretin levels and, subsequently, promote insulin release and suppress the glucagon level, resulting in lowered blood glucose levels. The first drug approved by the US Food and Drug Administration (FDA) was sitagliptin in 2006 for DM-II treatment. Later, further development of such class of drugs was also successful and approved for administration. To date, at least 10 DPP4-inhibitors have gained approval for clinical use. Among them, inhibitors that are recommended by international and national guidelines, including sitagliptin, vildagliptin, saxagliptin, linagliptin and alogliptin, were extensively studied, especially regarding their impact on malignancy outcome [6].

In addition to the promising effects in DM-II treatment, increasing studies have examined the role of DPP4-inhibitors in cancer development and treatment. Though there were concerns about the use of DPP4-inhibitors with an increased risk for pancreatic cancer, subsequent studies have revealed that there are only limited effects of DPP-4 inhibitors on pancreatic cancer and general cancer risk [7,8]. DPP4, also known as a cluster of differentiation 26 (CD26), is a cell membrane protein enzyme that promotes the degradation of growth factors and chemokines via its peptidase activity, removing dipeptides from the targets [9]. Such peptidase can be detected in a wide range of locations, including various tissues, blood and body fluids. Regarding tumorigenesis, CD26 acts as either a tumor suppressor or activator depending on the tumor location, its expression level and interaction with the microenvironment and chemokine targets [9,10,11]. Our previous work demonstrated a subpopulation of CD26(+) cancer stem cells (CSCs) uniformly present in both the primary and metastatic tumors in CRC patients with liver metastasis and revealed that the presence of CD26(+) CSCs in primary tumors predicted distant metastasis, which led to the development of distant metastasis in a mouse model and was associated with invasiveness and chemoresistance [12]. Studies, including ours, also showed that high CD26 expression in CRC specimens was associated with higher TNM staging, degree of differentiation and development of metastasis and was a predictor of poor prognosis after resection of CRC [13,14,15]. These observations suggest that CD26 is a potential therapeutic target for treating CRC.

The in vivo effect of a DPP4-inhibitor on CRC has been investigated in a few studies. Angelo et al. showed that sitagliptin, the most common DPP4-inhibitor, given chronically to DMH (a potent colon carcinogen)-induced rats reduces precancerous lesions in the colon and blood ROS, suggesting its anti-tumorigenesis effect [16]. Jang et al. also presented the suppression of mouse colorectal lung metastases development using DPP4-inhibitor [17]. However, another study by Wang et al. suggested DPP4-inhibitors enhanced tumor metastasis of colorectal cancer cells in nude mice model [18]. The major difference between the studies with contradicting conclusions was the use of the mouse model, in which the former two used immunocompetent rats/mice, whereas immunodeficient mice were utilised in the latter study. These findings suggested that the immune system status of the patients may affect the therapeutic response of DPP4-inhibitors, which warrants further investigation.

Hence, this study plans to investigate the potential therapeutic potential of DPP4-inhibitor on CRC patients by comparing the prognosis of post-operative CRC patients with diabetes complications and treated with DPP4-inhibitor to those treated with metformin and investigate the association with immune cell profile.

## 2. Materials and Methods

### 2.1. Subjects

CRC patients who had undergone curative surgery with prescription of DPP4-inhibitors history between January 2006 and December 2015 were included for investigating the therapeutic effect of DPP4-inhibitors. Patients were prescribed DPP4-inhibitors, including alogliptin, linagliptin, saxagliptin, sitagliptin and vildagliptin. Patients who had not been followed up for at least 5 years are excluded, except for those showing signs of recurrence or death within the follow-up period. CRC patients underwent curative surgery with a prescription of metformin, and no history of DPP4-inhibitors was included as the control group. The data collection protocol has been approved (UW 21-359) by the Institutional Review Board (IRB) of the University of Hong Kong.

### 2.2. Data Retrieval from Hong Kong Clinical Data Analysis and Reporting System (CDARS)

A population-based retrospective cohort was assembled from the Hong Kong Hospital Authority (HA) administrative database in the Hong Kong adult diabetes population from 1 January 2006 to 31 December 2015. CDARS has been extensively used for conducting high-quality large population-based studies [19]. The database contains comprehensive CRC patient (ICD-9-CM Diagnosis Code: 153.0–153.9; 154.0–154.3; 154.8) demographic and clinical characteristics include the year of diagnosis, age, sex, race/ethnicity, tumor location, tumor size, tumor stage, receipt of cancer treatment (such as surgery, chemotherapy, and radiation therapy); patient’s immune cell profile includes lymphocyte, neutrophil, neutrophil-to-lymphocyte ratio (NLR), platelet, platelet-to-lymphocyte ratio (PLR), monocyte, lymphocyte-to-monocyte ratio (LMR), albumin, prognostic nutritional index (PNI); HA drug prescription database contains information, including the date of drug dispensing, dosage unit, and quantity of drug dispensed, which was recorded. The baseline date of eligible patients was defined as the date of curative resection of primary CRC. Patients were observed from the baseline date until the incidence of event outcome, death from any cause, and censored at the last healthcare service utilization date, whichever came first.

### 2.3. Statistical Analysis

Univariate analysis of the association between recurrence or no recurrence and the treatment received was performed using Fisher’s exact test. The hazard ratios (HR) of the study and control groups and their 95% CIs were estimated from univariate or multivariate Cox Proportional Hazard models with disease-free survival and overall survival as the outcome. Survival curves were generated using the Kaplan–Meier test and compared by the log-rank test. The difference in immune cell profile between treatment and control group was compared by t-test, paired-test or Mann–Whitney U test. The criterion for statistical significance was a *p* < 0.05. All statistical analyses are conducted using SigmaPlot version 10.0 (Systat Software Inc., San Jose, CA, USA) and SPSS version 10.0 (SPSS Inc., Chicago, IL, USA).

## 3. Results

### 3.1. DPP4-Inhibitors Improve Prognosis of Post-Operative CRC Patients

**Figure 1:**
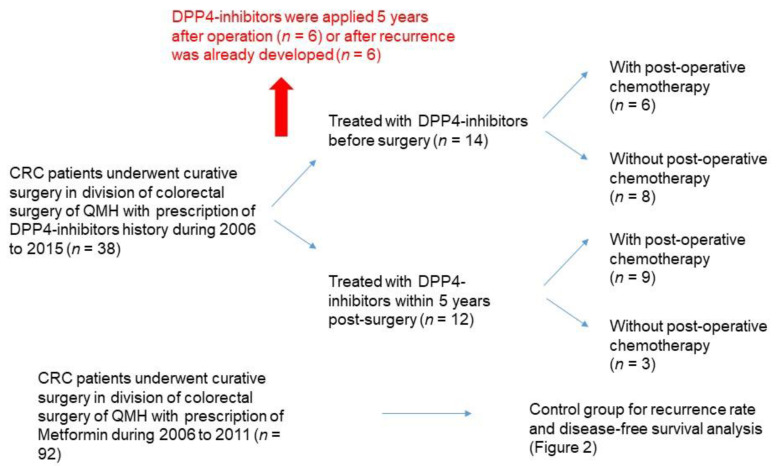
Patient selection in the study. CRC, colorectal cancer; DPP4, Dipeptidyl Peptidase 4; QMH, Queen Mary Hospital.

**Figure 2:**
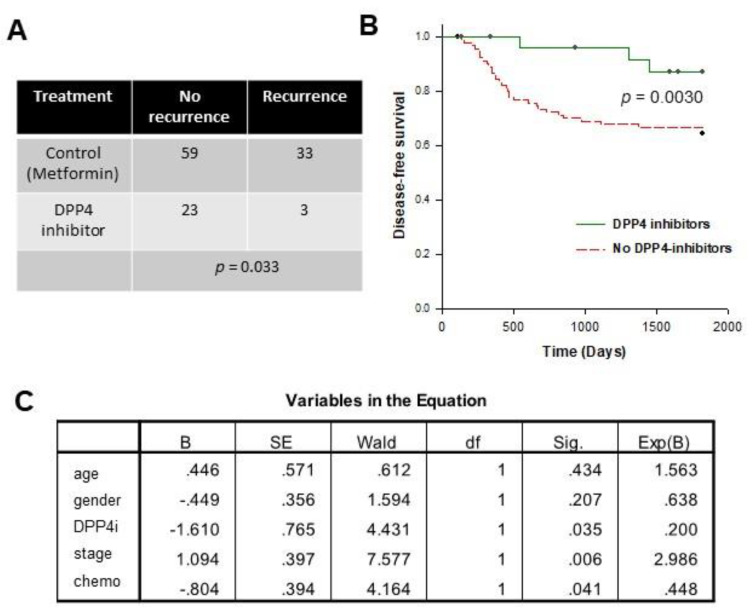
DPP4-inhibitors improve the prognosis of post-operative CRC patients. (**A**) There was a significantly lower rate of recurrence in DPP4-inhibitor-treated post-operative CRC patients when compared to the control group (metformin). (**B**) The DPP4-inhibitor-treated post-operative CRC patients showed significantly better disease-free survival when compared to the control group (metformin). (**C**) Cox regression analysis showed that DPP4-inhibitor and post-operative chemotherapy were favourable prognostic factors, whereas the high stage was an unfavourable prognostic factor for recurrence.

We retrieved the records of patients who received curative surgery in our department between 2006 and 2015, were DM-II patients and prescribed DPP4-inhibitors drugs. As shown in Figure 1, 39 patients were initially identified. We next screened out 13 patients for whom DPP4-inhibitors drugs were applied 5 years after operation (*n* = 6) or after recurrence was developed (*n* = 6). Metformin is one of the most commonly prescribed antidiabetic drugs used for the treatment of DM-II. Hence, for comparison, we retrieved the patient records who performed surgery in our department from 2006 to 2011, were DM-II patients and prescribed Metformin only (N = 92). The comparison of basic clinical parameters between the two groups was shown in Table 1. There was no significant difference in age and tumor stage between the two groups. On the other hand, there was a significantly higher proportion of male candidates in the DPP4-inhibitor group when compared to the metformin group, suggesting that it is more likely for male DM-II patients to switch to DPP4-inhibitor as 2nd line treatment. We compared the disease-free survival between these 2 groups of patients. The prognosis of the DPP4-inhibitor group was significantly better. Figure 2A showed that among the 92 patients in the metformin group, 33 of them showed recurrence within 5 years after surgery, whereas only 3 of the 26 patients in the DPP4-inhibitor group showed recurrence (*p* = 0.033). Log-rank analysis (Figure 2B) also demonstrated that the DPP4-inhibitor group also demonstrated significantly better disease-free survival (median DFS = 1733 days, 95% CI = 1596–1870 days) when compared to the control group (*p* = 0.030, median DFS = 1382 days, 95% CI = 1246–1518 days).

Furthermore, we performed Cox regression analysis to determine the independent variable (age, gender, stage, post-operative chemotherapy and DPP4-inhibitor application) for recurrence. As shown in Figure 2C, DPP4-inhibitor application is a favorable factor associated with a lower risk of recurrence (Hazard ratio = 0.200, *p* = 0.035). Post-operative chemotherapy was another favorable prognostic factor (Hazard ratio = 0.448, *p* = 0.041), whereas stage was an unfavorable prognostic factor for recurrence (Hazard ratio = 2.986, *p* = 0.006).

### 3.2. DPP4-Inhibitors Alter the Immune Cell Profile of CRC Patients which Associates with a Better Prognosis

**Figure 3:**
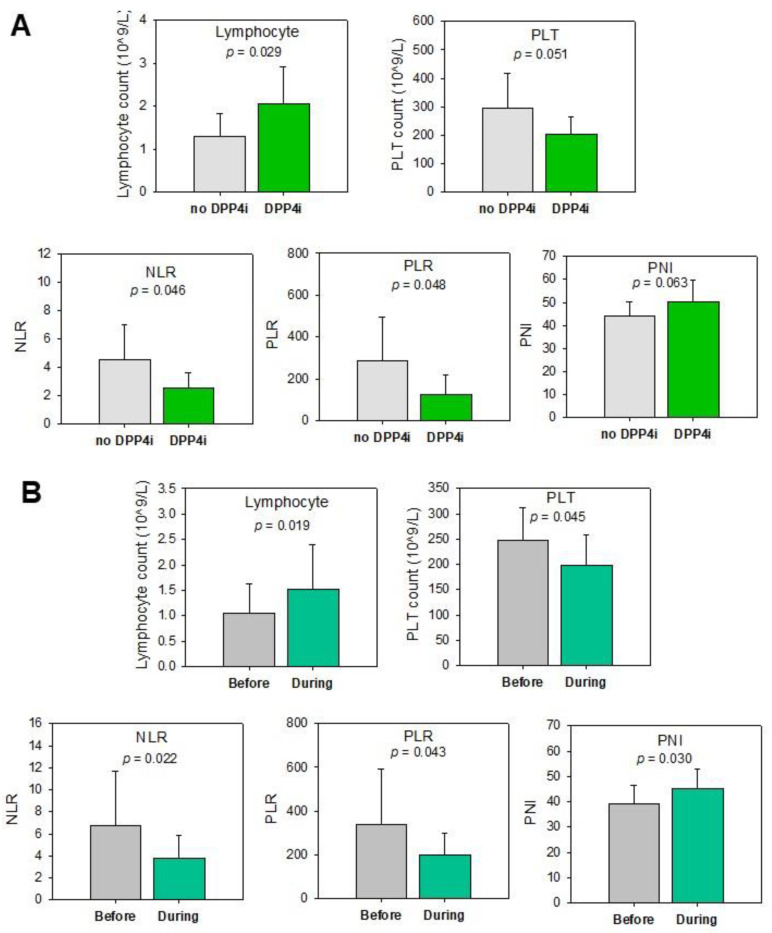
DPP4-inhibitors alter the immune cell profile of CRC patients. (**A**) The DPP4-inhibitor group showed a higher lymphocyte count, PNI and a lower PLT count, NLR and PLR in post-operative chemotherapy treated patients. (**B**) Change in the immune cell profile before and after DPP4-inhibitor treatment in post-operative patients. DPP4i: DPP4-inhibitors; Before: before DPP4-I treatment; During: administrated DPP4-inhibitors for 1–3 months (except over 3 months for PLT). PLT, platelet; NLR, neutrophil-to-lymphocyte ratio; PLR, platelet-to-lymphocyte ratio; PNI, prognostic nutritional index.

The immune system plays the most vital role in protection against diseases, including cancer. In addition to the prevention of tumor initiation and progression of stages, studies also suggested their involvement in driving tumor proliferation, invasion and metastasis, as well as their importance in treatment [20]. Since DPP4 is expressed predominantly on immune cells, and as mentioned above, previous studies demonstrated opposite effects of DPP4-inhibitors between immunocompetent mice and immunodeficient mice, we hypothesized the effect of DPP4-inhibitors on immune cells is one of the mechanisms associated with the improved prognosis. Hence, we compared the immune cell profile, including levels of lymphocyte, neutrophil, platelet (PLT), monocyte, albumin and neutrophil-to-lymphocyte ratio (NLR), platelet-to-lymphocyte ratio (PLR), lymphocyte-to-monocyte ratio (LMR) and prognostic nutritional index (PNI) in pre-operative and post-operative blood between CRC patients with and without DPP4-inhibitor treatment. Our pilot data showed that CRC patients with DPP4-inhibitor treatment showed a trend of higher post-operative lymphocyte count (*p* = 0.096, data not shown) when compared to the control group. In addition, as shown in Figure 3A, in patients treated with post-operative chemotherapy, the DPP4-inhibitor group showed a significantly higher lymphocyte count (*p* = 0.029), lower NLR (*p* = 0.046) and PLR (*p* = 0.048). A trend of lower PLT (*p* = 0.051) and higher PNI (*p* = 0.063) was also observed in the DPP4-inhibitor group. These changes had been reported to be a good prognostic factor for CRC patients’ disease-free and overall survivals [21,22]. Hence, these findings provided further evidence to our hypothesis that DPP4-inhibitor is a potential therapeutic approach for treating CRC patients.

To further confirm the change in lymphocyte count, NLR, PLR, PLT and PNI in the DPP4-inhibitor group was indeed caused by the effect of DPP4-inhibitors, we compared these parameters before and after DPP4-inhibitor treatment in post-operative patients (Figure 3B). Our results showed that after DPP4-inhibitor treatment (1–3 months), there was a significant increase in lymphocyte count (*p* = 0.019) and PNI (*p* = 0.030), decrease in NLR (*p* = 0.022) and PLR (*p* = 0.043). The level of PLT was also decreased after prolonged treatment (>3 months) of DPP4-inhibitors (*p* = 0.045). These results suggested that DPP4-inhibitor causes changes in immune cell profile, which is associated with a better prognosis of post-operative CRC patients.

### 3.3. Immune Cell Profile of CRC Patients is a Potential Biomarker for Response to DPP4-Inhibitor Treatment

**Figure 4:**
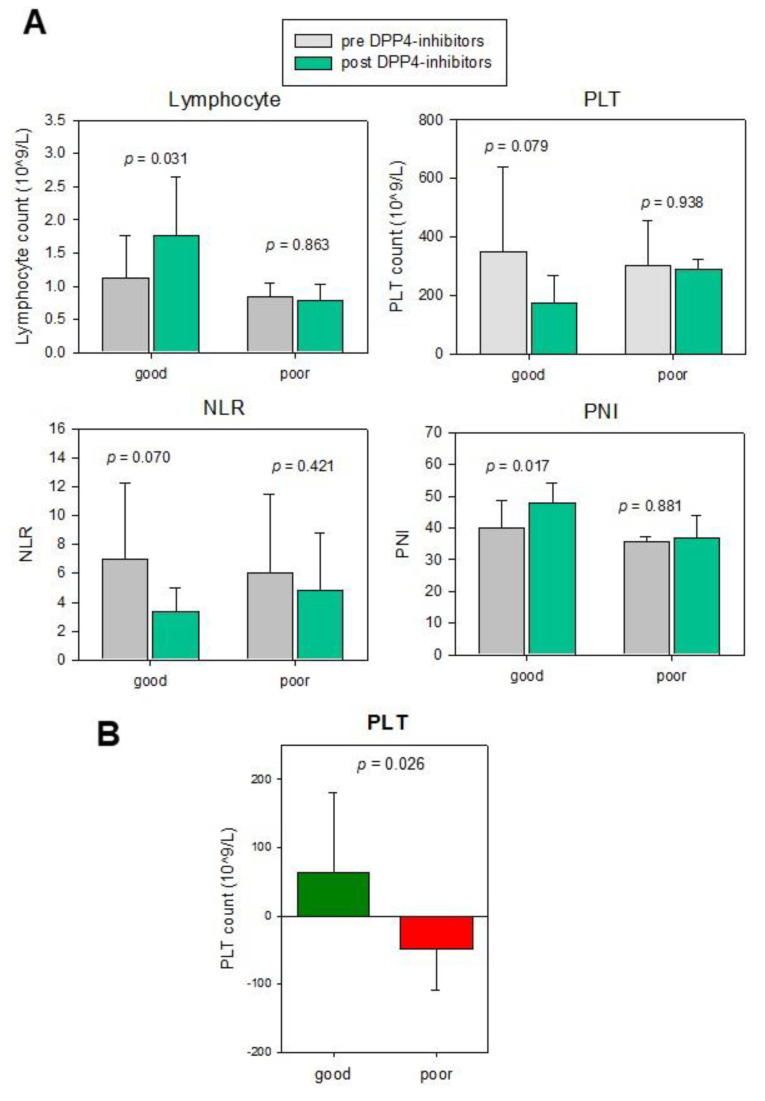
The immune cell profile of CRC patients as a potential biomarker for efficacy of DPP4-inhibitor treatment. (**A**) Changes of lymphocytes, PLT, NLR and PNI before and after DPP4-inhibitors are significant in good prognosis patients but insignificant in the poor prognosis group. (**B**) Change of post vs. pre-operation PLT count was significantly associated with DPP4-inhibitor efficacy.

We hypothesized the change of these parameters after DPP4-inhibitor treatment could be a potential prognostic biomarker for the CRC patients, hence compared these parameters before and after DPP4-inhibitor treatment in these CRC patients with good (*n* = 8) or poor overall survival (*n* = 2). As shown in Figure 4A, our results suggested a significant or close-to-significant increase in lymphocyte count (*p* = 0.031) and PNI (*p* = 0.017) and decrease in NLR (*p* = 0.070) and PLR (*p* = 0.079) in good prognosis patients, whereas the change in the poor prognosis group was insignificant, suggesting that the change in these immune cell parameters can early predict the efficacy of DPP4-inhibitor on the prognosis of post-operative patients.

Whether the immune cell profile can identify CRC patients suitable for DPP4-inhibitor treatment was also examined. Hence we compared the pre-operative and post-operative immune cell profile in good and poor prognosis groups upon DPP4-inhibitors treatment. The results illustrated that the change of post vs. pre-operation PLT count was significantly associated with DPP4-inhibitor efficacy (Figure 4B, *p* = 0.026). The good prognosis group showed an increase in PLT count (mean = 63.4), whereas the poor prognosis group showed a reduction (mean = −48.6). This suggests changes in post-operative vs. pre-operative PLT level are a potential biomarker for selecting patients who will show a favorable prognosis upon DPP4-inhibitor treatment.

## 4. Discussion

Colorectal cancer (CRC) is one of the most common cancers and the leading cause of cancer deaths worldwide. The poor prognosis for patients, in particular, those at advanced stages, highlights the urgent need for more effective treatments. Our co-investigator of this study previously performed an original study in Hong Kong to evaluate the risks of cancer among patients with DM-II on Metformin dual therapy with inadequate control and were synchronously administered with third-line glucose-reducing medications, namely DPP4-inhibitors, insulin or thiazolidinediones [19]. In the study, researchers suggested the correlation between DPP4-inhibitor administration and reduced risks of cancer and cancer mortality, whereas the overall mortality, regardless of the cause, remains consistent. Based on those findings, we investigated whether DPP4-inhibitors may be used in the management of colorectal cancer. Since some colorectal patients with DM-II were treated with DPP4-inhibitors, these patients can be classified as a model to investigate the therapeutic effect of DPP4-inhibitors by comparing such group to the colorectal patients with DM-II yet no DPP4-inhibitors prescription.

Concurrently during our pilot study, two researchers investigated the therapeutic effect of DPP4-inhibitors on CRC patients. Azka et al. in 2018 reported a multi-centre retrospective analysis investigating the correlation between DPP4-inhibitors administration and progression-free survival (PFS) in diabetic patients with CRC or advanced airway cancer [23]. They suggested a promoted PFS in DPP4-inhibitor-prescribed groups in comparison to control subjects, with hazard ratio = 0.42 (95% CI: 0.21–0.84) and *p* = 0.014. Another national database study showed that the favorable effect of DPP4-inhibitors on CRC patients did not reach the statistical significance (HR of 0.87; CI: 0.75–1.00, *p* = 0.055), while significant improvement in survival was achieved by administration of synergic DPP4-inhibitors and metformin (HR of 0.77; CI: 0.67–0.89, P = 0.003) [24].

**Figure 5:**
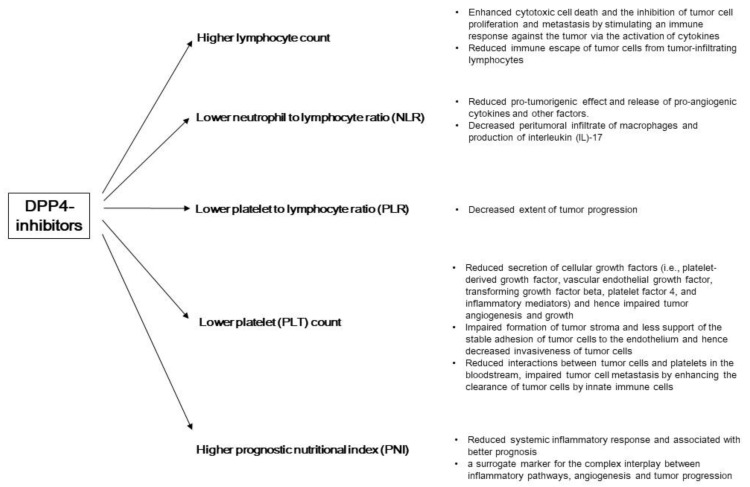
A summary of the findings from this study on the changes in certain immune cells and systemic inflammatory responses in CRC patients treated with DPP4-inhibitors and the subsequent mechanisms associated with better prognosis.

Though the therapeutic effects of DPP4-inhibitors have been suggested by previous studies, concerns before concluding the potential clinical usage of DPP4-inhibitors still exist. First, the therapeutic effects of DPP4-inhibitors on CRC patients mentioned above were analyzed using the US population, but the effect of DPP4-inhibitors on the Chinese population is unknown, which will also serve as the reference for other Asian countries. Secondly, though DPP4-inhibitors show some encouraging data in improving the prognosis of CRC patients, the mechanism involved is still poorly understood. This study’s findings suggest that DPP4-inhibitor can improve the prognosis of CRC patients in our region, and one possible explanation of its therapeutic effect is through altering the immune system. Figure 5 summarized this study’s finding on the changes in certain immune cells and systemic inflammatory responses in CRC patients treated with DPP4-inhibitors and the subsequent mechanisms associated with better prognosis.

There is evidence demonstrating the potential modulatory functions of DPP4 in the immune system and its association with tumor initiation and progression. DPP4, also known as the lymphocyte cell surface protein CD26, is widely expressed in many types of immune cells and regulates their functions, including CD4(+) and CD8(+) T cells, B cells, natural killer cells, dendritic cells and macrophages [25]. In addition, DPP4 has a variety of substrates, including incretin hormones, cytokines, chemokines, neuropeptides and growth factors [26]. One of the well-known tumor progression effects of DPP4 is through its catalytic activity on chemokines. The success of antitumor immune responses depends on the infiltration of effector T cells into solid tumors, which is guided by chemokines, whereas DPP4 limits lymphocyte migration to sites of inflammation and tumors through post-translational processing of chemokines [27]. On the other hand, several studies demonstrated that inhibition of DPP4 possessed an antitumor effect through inhibiting the effect of DPP4 on chemokines and thus activity of immune cells. For example, the inhibition of DPP4 enzymatic activity in melanoma-enhanced tumor rejection by preserving biologically active CXCL10 and increasing trafficking into the tumor by lymphocytes expressing the counter-receptor CXCR3 [27]; In breast and liver cancer, DPP4-inhibitor sitagliptin treatment resulted in a higher level of the chemokine CCL11 and increased migration of eosinophils into solid tumors, which in turn enhanced degranulation-dependent control of tumor growth [28]; In a preclinical model of CRC study, DPP4 inhibition resulted in a marked delay of CT26 tumor growth, which correlated with enhanced infiltration of lymphocytes, including T cell subsets and natural killer cells [27]. Our study further revealed that DPP4-inhibitor-treated CRC patients demonstrated changes in post-operative lymphocyte count, NLR, PLR, PLT and PNI, which were features associated with better prognosis. Hence, further investigation is warranted to evaluate the antitumor effect of DPP4-inhibitors on CRC. Moreover, since DPP4-inhibition is associated with enhanced antitumor immune response, their potential to enhance the treatment effect of immunotherapy warrants further investigation. In fact, the preclinical CRC model has suggested that DPP4 inhibition improved adjuvant-based immunotherapy, adoptive T cell transfer and checkpoint blockade [27].

In addition, DPP4 binds to several molecules to trigger modulation of immune responsiveness. These non-catalytic functions are exerted via binding of DPP4 to binding partners such as adenosine deaminase (ADA), CXC chemokine receptor 4 (CXCR4), caveolin-1, CD45, CARMA1(CARD11), M6P/IGFRII, Thromboxane A2 receptor, HIV-TAT and HIV-gp120 (reviewed in [29]). In particular, the interaction between DPP-4 and ADA and subsequent signal transduction are most extensively investigated. ADA is able to catalyze the hydrolytic deamination of adenosine to inosine, whereas a high concentration of adenosine impaired T lymphocytes proliferation. Only the DPP4-bounded ADA is functional and could counteract the inhibitory effect of extracellular adenosine. This DPP4/ADA/adenosine pathway is suggested to be crucial for T-cell activation [29].

In this study, the three patients in the DPP4-inhibitor groups developing recurrence were treated with sitagliptin 100 mg daily, sitagliptin 50 mg daily or linagliptin 5 mg daily. However, as the sample size was also bigger in these three dosage groups (Table 1), we think that there was no significant difference in therapeutic performance among types and dosages of DPP4-inhibitors for improving the prognosis of CRC patients. For clinical trials in the future, we suggest that the DPP4-inhibitor treatment for CRC patients could be started with the routine clinical dosage, but a higher dosage can be considered if the patients do not respond well to the treatment based on pre-/post-treatment changes in lymphocyte count, PNI, NLR, PLR and PLT count as we mentioned in Section 3.3.

Drug repurposing is one attractive approach for a novel therapeutic strategy for cancer patients since it requires a long period for new drugs to undergo series of preclinical and clinical trials before they can be approved to be used on CRC patients. This study investigated the therapeutic effect of already approved drugs, DPP4-inhibitors, which are commonly prescribed to treat diabetes. Data were available from several studies, and our preliminary data demonstrated their therapeutic effect on improving the prognosis of CRC patients. We are expecting more studies to validate the efficacy of DPP4-inhibitors in treating CRC patients or subgroups of CRC patients (such as those with diabetes comorbidity), and therefore, they can be promptly entered clinical application to improve the prognosis of CRC patients.

## 5. Conclusions

This study demonstrated the association of DPP4-inhibitor treatment and better prognosis of CRC patients. Post-operative patients treated with DPP4-inhibitors showed a significantly enhanced 5-year disease-free survival when compared to metformin-treated counterparts, and DPP4-inhibitor application is a favorable factor associated with a lower risk of recurrence. Furthermore, our results suggested that the immune cell profile of CRC patients is a potential biomarker for stratifying patients who will show a better response to DPP4-inhibitor treatment.

## Figures and Tables

**Table 1:** Clinical parameters of patients involved in this study. * The tumor stage data of 1 patient in DPP4-inhibitor group and 1 patient in control group is unknown.

	DPP4-Inhibitor Group	Control Group (Metformin)
**Gender**		
Male	22	47
Female	4	45
	*p* = 0.005	
	
**Age**		
Above 65	20	69
Below or = 65	6	23
	*p* = 0.955	
	
Tumor stage *		
Low	15	50
High	10	41
	*p* = 0.823	
	
**Single or combined DM-II treatment**		
Single	11	92
Combined (with Metformin)	15	
		
**DPP4-inhibitor/dosage**		
Sitagliptin/50 mg daily	6	
Sitagliptin/100 mg daily	7	
Linagliptin/5 mg daily	7	
Vildagliptin/50 mg daily	3	
Vildagliptin/50 mg bi-daily	2	
Saxaglitptin/5 mg daily	1	

## Data Availability

The data presented in this study are available in the article.
